# Network pharmacology and molecular docking reveal the effective substances and active mechanisms of Dalbergia Odoriferain protecting against ischemic stroke

**DOI:** 10.1371/journal.pone.0255736

**Published:** 2021-09-28

**Authors:** Kedi Liu, Xingru Tao, Jing Su, Fei Li, Fei Mu, Shi Zhao, Xinming Lu, Jing Li, Sha Chen, Taiwei Dong, Jialin Duan, Peifeng Wei, Miaomiao Xi

**Affiliations:** 1 College of Pharmacy, Shaanxi University of Chinese Medicine, Xianyang, Shaanxi, China; 2 TANK Medicinal Biology Institute of Xi’an, Xi’an, Shaanxi, China; 3 Department of Pharmacy, Tangdu Hospital, Fourth Military Medical University, Xi’an, Shaanxi, China; 4 Department of Pharmacy, Xijing Hospital, Fourth Military Medical University, Xi’an, Shaanxi, China; 5 YouYi Clinical Laboratories of Shaanxi, Xi’an, Shaanxi, China; 6 Institute of Medical Research, Northwestern Polytechnical University, Xi’an, China; 7 National Drug Clinical Trial Institute, The Second Affiliated Hospital, Shaanxi University of Chinese Medicine, Xianyang, Shaanxi, China; Universita degli Studi di Napoli Federico II, ITALY

## Abstract

Dalbergia Odorifera (DO) has been widely used for the treatment of cardiovascular and cerebrovascular diseasesinclinical. However, the effective substances and possible mechanisms of DO are still unclear. In this study, network pharmacology and molecular docking were used toelucidate the effective substances and active mechanisms of DO in treating ischemic stroke (IS). 544 DO-related targets from 29 bioactive components and 344 IS-related targets were collected, among them, 71 overlapping common targets were got. Enrichment analysis showed that 12 components were the possible bioactive components in DO, which regulating 9 important signaling pathways in 3 biological processes including ‘oxidative stress’ (KEGG:04151, KEGG:04068, KEGG:04915), ‘inflammatory response’(KEGG:04668, KEGG:04064) and ‘vascular endothelial function regulation’(KEGG:04066, KEGG:04370). Among these, 5 bioactive components with degree≥20 among the 12 potential bioactive components were selected to be docked with the top5 core targets using AutodockVina software. According to the results of molecular docking, the binding sites of core target protein AKT1 and MOL002974, MOL002975, and MOL002914 were 9, 8, and 6, respectively, and they contained 2, 1, and 0 threonine residues, respectively. And some binding sites were consistent, which may be the reason for the similarities and differences between the docking results of the 3 core bioactive components. The results of *in vitro* experiments showed that OGD/R could inhibit cell survival and AKT phosphorylation which were reversed by the 3 core bioactive components. Among them, MOL002974 (butein) had a slightly better effect. Therefore, the protective effect of MOL002974 (butein) against cerebral ischemia was further evaluated in a rat model of middle cerebral artery occlusion (MCAO) by detecting neurological score, cerebral infarction volume and lactate dehydrogenase (LDH) level. The results indicated that MOL002974 (butein) could significantly improve the neurological score of rats, decrease cerebral infarction volume, and inhibit the level of LDH in the cerebral tissue and serum in a dose-dependent manner. In conclusion, network pharmacology and molecular docking predicate the possible effective substances and mechanisms of DO in treating IS. And the results are verified by the *in vitro* and *in vivo* experiments. This research reveals the possible effective substances from DO and its active mechanisms for treating IS and provides a new direction for the secondary development of DO for treating IS.

## 1. Background

Stroke is the third leading death cause worldwide, which seriously threat human health. It is divided as ischemic stroke (IS) and hemorrhagic stroke (HS), of which IS accounts for more than 75% [1]. The possible mechanisms of IS includes oxidative stress, inflammation, apoptosis, energy metabolism disorders, etc [2, 3]. In clinical, thrombolytic agents are the commonly used drugs in treating IS, however the arrow time window limits its application [4]. Therefore, it is urgent to find novel drugs to treat IS.

Dalbergia Odorifera (DO; Chinese name, Jiangxiang) with the effects of promoting circulation and removing blood stasis, is an important composition of Huoxue Tongmai Capsule and Guanxin Danshen Capsule which were used in treating cardiovascular and cerebrovascular diseases [5]. The mainly chemical constituents of DO are flavonoids and volatile oil [6–8], which have effects of anti-oxidation [9–12], anti-inflammatory [13], and vascular endothelial function regulation [14–16]. In precious study, the effects of DO on IS had been well studied, however, the effective substances and active mechanisms were largely unknown to us, which limits the exploitation and application of this herb.

Herbs always contains many components (dozens or hundreds compounds), so it is difficult to study their effective substances and mechanisms using traditional methods. Network pharmacology is a novel method that combines systematic network analysis and pharmacology, which always used to clarify the synergistic effects and potential mechanisms of components-component networks, components-target networks, and targets-disease networks at the molecular level, so as to understand the interaction relationships among components, genes, proteins, and diseases [17]. Molecular docking is a computer-aided drug design technology, which uses computer technology to simulate the geometric structure and intermolecular interaction force of molecules through stoichiometric calculation methods to find the best binding mode of small molecule drugs and known structural macromolecules (proteins) [18]. Network pharmacology and molecular docking can study many chemicals and targets in the meantime, so they may provide possible research approaches for the TCM studying.

**Fig 1:**
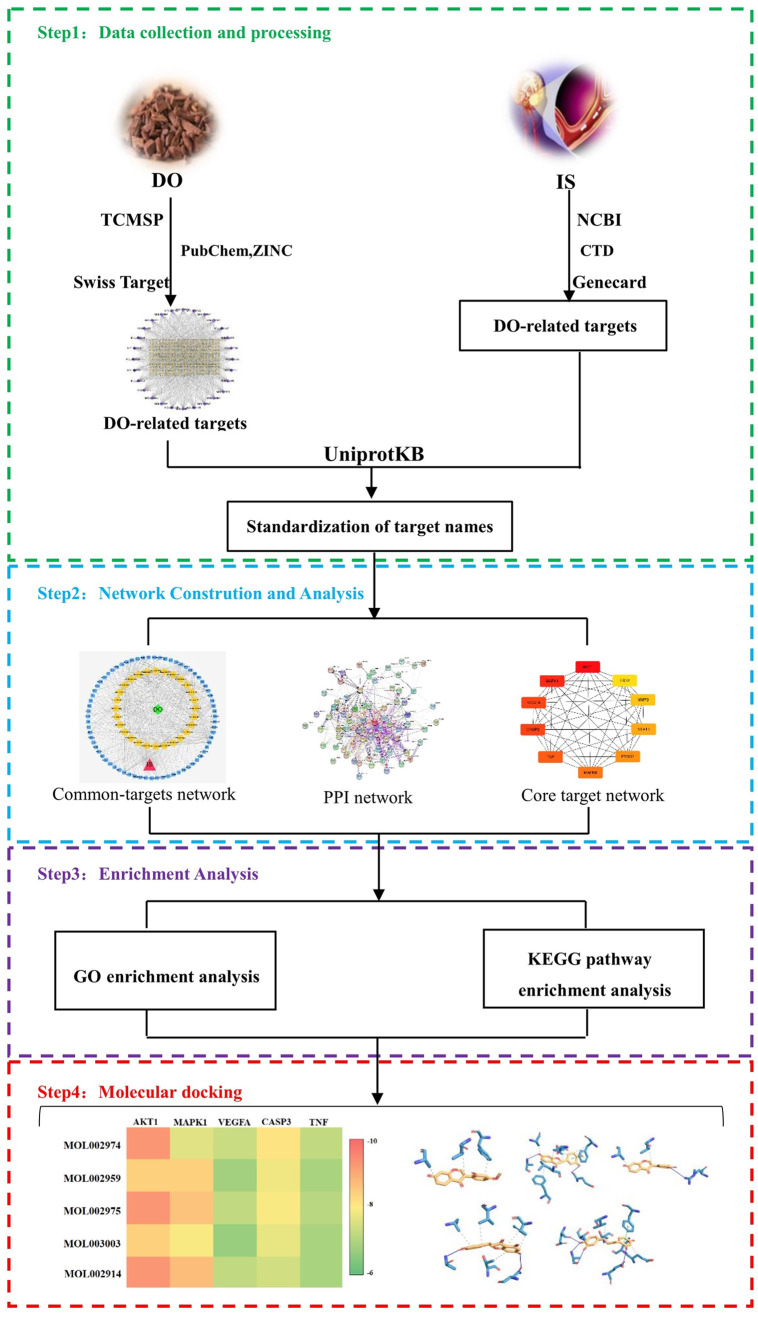
Study flowchart of network pharmacology and molecular docking.

In this study, to illustrate the effective substances and possible mechanisms of DO, network pharmacology and molecular docking were used. The bioactive components from DO were screened, and the core targets of them were analyzed. Next, we constructed the multi-level interaction network of ‘component-target-pathway’, and explored the characteristics of the combination between chemicals and targets. Finally, *in vitro* experiments were used to verify these results. The study flowchart of network pharmacology and molecular docking is displayed in Fig 1.

## 2. Methods

### 2.1. Data collection and processing

#### 2.1.1. Composite components of DO

Data of the compounds of DO were mainly collected from the natural product databases for Chinese herbal medicine: Traditional Chinese Medicine Systems Pharmacology Database and Analysis Platform (TCMSP, http://ibts.hkbu.edu.hk/LSP/tcmsp.php).

#### 2.1.2. Screening of bioactive components

TCM is mainly used by oral administration. Therefore, the bioactive components of DO were screened by 2 main parameters affecting gastrointestinal absorption: oral bioavailability(OB)≥40% and drug-likeness(DL)≥0.18.

#### 2.1.3. Target prediction of bioactive components

Swiss Target Prediction (http://www.swisstargetprediction.ch/) is a web server based on 2D and 3D similarity measurement of known ligands, which can accurately predict the target of bioactive components. First, we obtained the molecular structure of bioactive components in DO and the structural formulas ‘canonical smiles’ and ‘sdf’ from PubChem (https://pubchem.ncbi.nlm.nih.gov/) and ZINC databases (http://zinc15.docking.org). Then we used the species as ‘Homo sapiens’ to predict the target through the Swiss Target Database, and used UniProt knowledge database (https://sparql.uniprot.org/) to standardize the target name and removed the duplicate target.

#### 2.1.4. IS-related targets

We searched the keyword ‘stroke’ in the GeneCards database (Gene, https://www.genecards.org/) and the National Centre for Biotechnology Information Gene (NCBI, https://www.ncbi.nlm.nih.gov/gene/). Also, we searched keywords such as ‘stroke’ and ‘cerebral infarction’ in the Comparative Toxicogenomics Database (CTD, http://ctdbase.org/). Then, the results of the 3 databases were summarized and the duplicates were deleted.

### 2.2. Network construction

#### 2.2.1. Common-target network construction

We screened the common targets of DO bioactive components and IS, and used Cytoscape (http://www.cytoscape.org) to build a common target network.

#### 2.2.2. PPI network construction

PPI network as a new drug research method can be used to clarify the relationship between the predicted targets and other human proteins. The String (https://string-db.org/) is a database for searching protein interactions, which provides information on protein prediction and experimental interactions. The PPI network of common targets of IS-related and DO-related targets were constructed by String database and the visualization of the PPI network was achieved by Cytoscape 3.7.1 software.

### 2.3. Core target prediction

A topological analysis was performed on the network to screen out the core targets of top10 by using the cytoHubba. The more darker of the color means the more targets connected to it and the closer connection.

### 2.4. Screening the potential bioactive components

A topological analysis was performed on the network by using Analyze Network to sort according to the size of the node Degree, and to screen out the potential bioactive components according to ‘Degree> Median’.

### 2.5. Enrichment analysis

We used ‘*P*≤0.05 using the Bonferroni correction’ as the screening condition to perform GO enrichment analysis on the network through the DAVID database. Then, with ‘*P*≤0.05’ as the screening condition, we used the ClueGO software to analyze the KEGG channel enrichment of the network.

### 2.6. Molecular docking

We downloaded the target protein structures from the RSCB PDB database (http://www.rcsb.org/), and used PyMOL software to remove the crystalline water and other small molecules of each protein structure, and saved it as pdb format. Then we imported the structure files into the AutodockTool 1.5.6 program, added the atomic charge, and saved it as pdbqt format after adding hydrogenation. Next, the mol2 format file of the bioactive components were imported into the AutodockTool1.5.6 program, and added the atomic charge, then saved it as the pdbqt format as the docking ligands. Autodock vina software was used to simulate molecular docking to determine the binding affinity of the target proteins and the potential bioactive components [19]. The binding affinities between these bioactive components and the target proteins were used as the evaluation criteria. The smaller the binding affinity, the better the docking is.

### 2.7. Experimental verification

#### 2.7.1 Materials

MOL002974 (butein), MOL002975 (butin), MOL002914 (Eriodyctiol) were purchased from Shanghai Yuanye Biotechnology Co., Ltd. (HPLC≥98%). Edaravone injection was purchased from Jilin Boda Pharmaceutical Co., Ltd.; PC12 cells were donated by the Department of Toxicology, Department of Preventive Medicine, Air Force Military Medical University. Sprague-Dawley rats (weight 250.0±5.0g) were provided by the Experimental Animal Center of the Fourth Military Medical University, and the production license number is SCXK (Shaanxi) 2019–001. All animals followed the relevant regulations of experimental animal ethics and passed the animal experiment ethics review of Shaanxi University of Traditional Chinese Medicine. Rats were reared in separate cages, with strict control of temperature (22±2°C), humidity (55%-75%), light (12-hour cycle) and other feeding conditions. Primary antibodies against GAPDH were purchased from Cell Signaling Technology (Danvers, MA, USA). Primary antibodies against AKT and p-AKT were purchased from ProteinTech Group, Inc. (Rosemont, IL, USA). Secondary antibody was bought from Abbkine (California, USA).

#### 2.7.2. Cell culture and treatment

PC12 cells were cultured by a DMEM high-glycemic medium containing 10% FBS and 1% bi-antibody in a constant temperature incubator with 37°C and 5% CO_2_. For oxygen-glucose deprivation, PC12 cells were cultured with glucose-free DMEM medium in an anaerobic incubator (95% N_2_, 5% CO_2_) at 37°C for 3 hours. For reperfusion, the culture medium was replaced with complete medium and cultured for another 24 hours, these process was defined as oxygen-glucose deprivation/reperfusion model (OGD/R). The control group was cultured in normal DMEM medium under normal culture conditions. After 3 hours of an aerobic incubation, the MOL002974 treatment groups were replaced with a complete medium containing 0.5, 1, and 2 μmol/L MOL002974 and cultured for 24 hours, the MOL002975 and MOL002914 treatment groups were replaced with a complete medium containing1, 2, and 4 μmol/L MOL002975, MOL002914 and cultured for 24 hours.

#### 2.7.3. Cell viability analysis

PC12 cells were seeded in a 96-well plate at a cell density of 5×10^4^ cells/well. After the cells adhered, they were grouped according to "2.7.2". After the culture, the medium was replaced with CCK8 medium and incubated in a 37°C, 5% CO_2_ cell incubator for 4 hours, and the absorbance value of each well was measured at a wavelength of 450 nm using a microplate reader.


$$\text{Survival}\;\text{rate}\left(\%\right)=({\text{OD}}_{\text{addition}\;\text{group}}-{\text{OD}}_{\text{blank}\;\text{group}})/({\text{OD}}_{\text{normal}\;\text{control}\;\text{group}}-{\text{OD}}_{\text{blank}\;\text{group}})\times 100\%.$$

#### 2.7.4. Western blot analysis

First, the cells were lysed with a pre-cooled RIPA lysate buffer containing protease inhibitors. The cell lysates were centrifuged at 12000 g for 10 min in 4°C, and the concentration of protein were determined by BCA method. The same amount of protein (20ug) was isolated with 10% SDS-PAGE and transferred onto the PVDF membrane. At room temperature, the membrane were blocked by 2h in TBST with 5% milk and incubated overnight at 4°C with primary antibodies. Then the membranes were incubated with secondary antibody at 37°C for 2h. The blots were visualized by ECL reagents, the bands were scanned and analyzed by quantitative-image analysis software.

#### 2.7.5. Animals grouping and treatment

The rats were randomly divided into 7 groups, with six rats in each. Sham rats received saline by intraperitoneal injection (20ml/kg) once a day for 3 consecutive days. Model rats received saline by intraperitoneal injection (20ml/kg) once a day for 3 consecutive days. Edaravone group rats (Eda group) received edaravone injection by intraperitoneal injection (3mg/kg) once a day for 3 consecutive days. Solvent control group rats (DMSO group) received DMSO by intraperitoneal injection (1.9mg/kg) once a day for 3 consecutive days. Butein high-dose group rats (butein-8 group) received butein by intraperitoneal injection (8mg/kg) once a day for 3 consecutive days. Butein medium-dose group rats (butein-4 group) received butein by intraperitoneal injection (4mg/kg) once a day for 3 consecutive days. Butein low-dose group rats (butein-2 group) received butein by intraperitoneal injection (2mg/kg) once a day for 3 consecutive days.

#### 2.7.6. Samples collection and processing

24 hours after the last injection of butein, the rats were anesthetized by intraperitoneal injection of 4% pentobarbital sodium.1ml of blood was taken from the abdominal aorta, left standing at room temperature for 30 minutes, centrifuged at 3500g for 3 minutes, and the supernatant (serum) was taken for LDH testing. The whole brain was removed, washed with pre-cooled 1×PBS, and dried with filter paper 3 whole brain of each group was used for cerebral infarction volume detection and 3 whole brain of each group was used for LDH level testing.

#### 2.7.7. Neurofunctional scores analysis

After 24 h of reperfusion, neurological deficits were evaluated by a blinded observer with a 5-point-scale scoring system as described previously. 0 = no obvious neurological deficit; 1 = inability to extend the contralateral forelimb; 2 = circle to the opposite side of ischemia; 3 = unable to bear the weight of the contralateral side; 4 = no voluntary movement or disturbance of consciousness.

#### 2.7.8. Cerebral infarct volume analysis

After neurological assessment, the rat was decapitated and the cerebral was taken out for the infarct volume measurement. The whole brain was sliced into uniform coronal slices, each slice 2 mm thick. The sections were stained with 1% 2,3,5-triphenyltetrazolium chloride (TTC), kept at 37°C for 10 minutes, and fixed in 4% paraformaldehyde buffer. For analysis, the slices were photographed with a digital camera. A computerized image analysis system was used to determine the infarct area of each slice. Infarct volume was expressed as percentages of contralateral hemispheric volume.

#### 2.7.9. Lactate dehydrogenase (LDH) levels analysis

The levels of LDH in serum and cerebral tissue were detected by using LDH assay kit following the manufacturer’s instruction. The data were measured by a microplate reader at 440 nm.

### 2.8. Statistical analysis

SPSS20.0 software was used to perform statistical analyses. The results were shown as $\overset{-}{X}\pm SD$. ANOVA followed by LSD-t test were used for mean comparison between groups, with *P*<0.05 indicating statistical significant.

## 3. Results

### 3.1. DO component-target network

**Fig 2:**
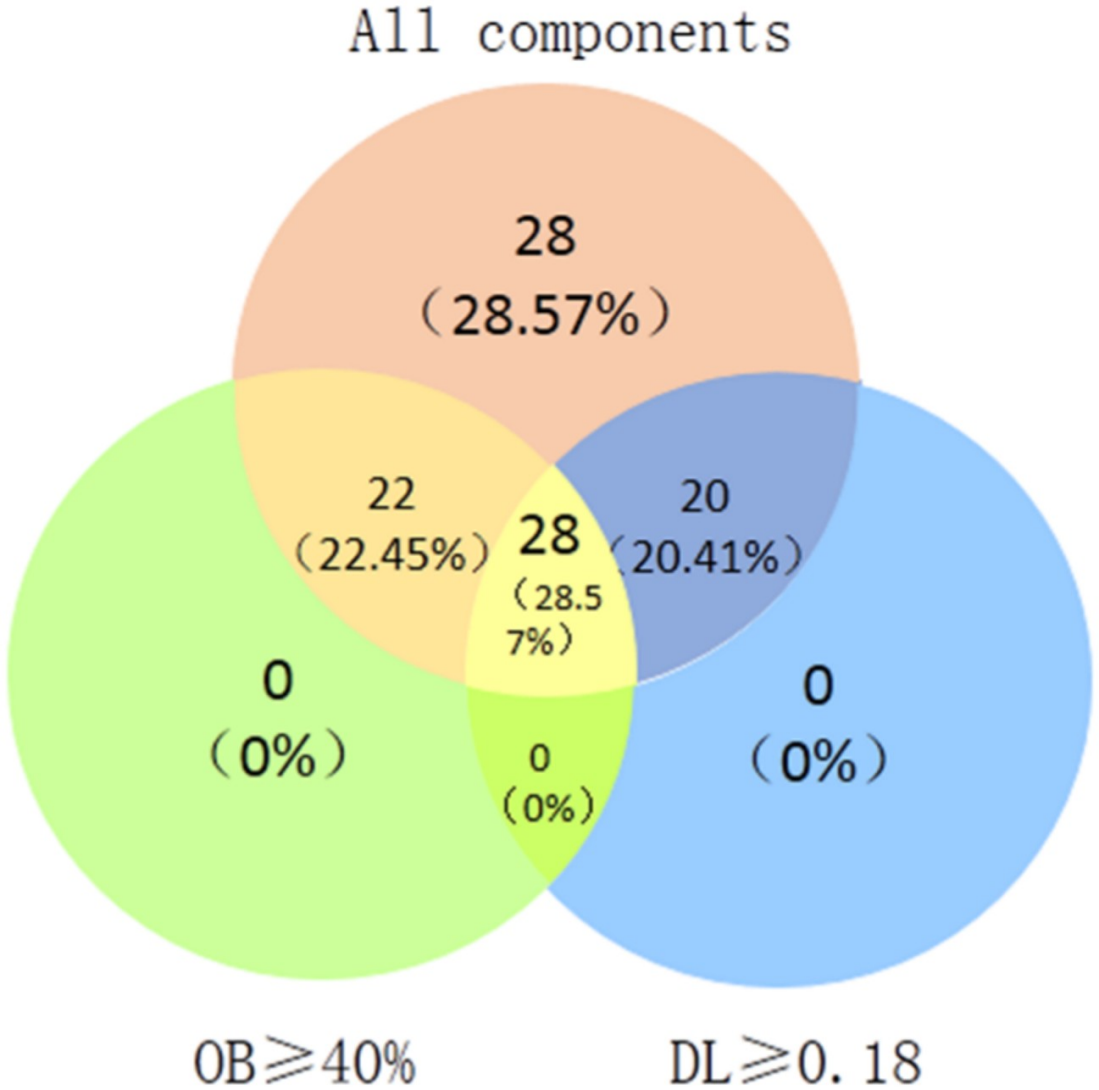
DO component-target network. Venn diagram: 98 components (pink section), and 28 bioactive components screened by two ADME-related models (green section stands for the components of OB≥40%, blue section stands for DL≥0.18).

98 components were collected in DO based on the TCMSP database, and 28 bioactive components were selected with the screening conditions of OB≥40% and DL≥0.18 (Fig 2). In previous and our preliminary studies, butein has protective effects against IS, so butein was selected in the further studies [20].

**Fig 3:**
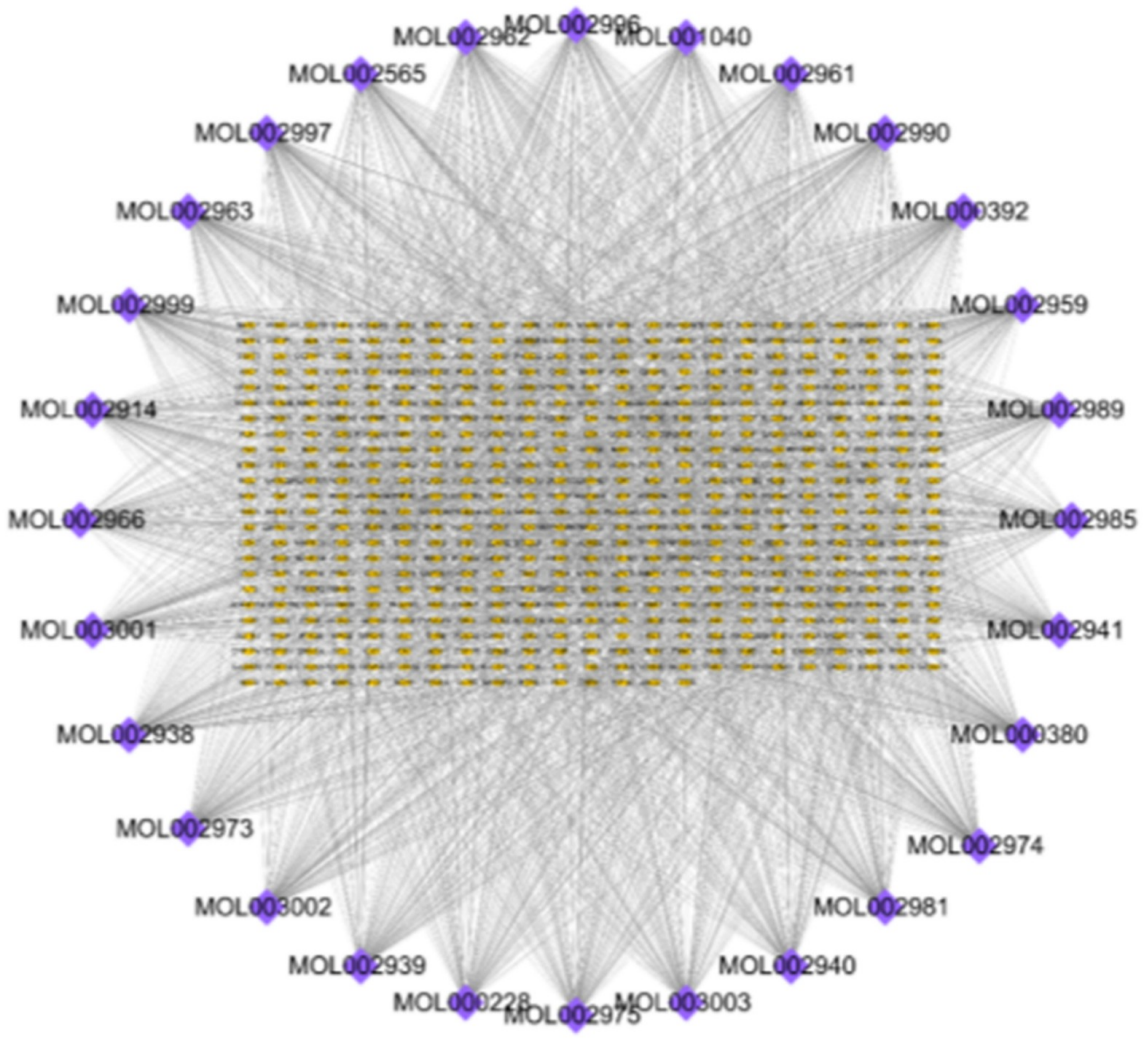
Construction of DO component-targets visual network, including 572 nodes and 2772 edges. Blue nodes stand for bioactive components from DO, yellow nodes stand for targets.

Next, we used the Swiss Target Prediction database to predict the target of 29 monomer structures, set the species to ‘Homo sapiens’, removed the duplicates and used UniProtKB to standardize the target name. A total of 544 targets were obtained. The component-target network was constructed by using Cytoscape software, which contained 572 nodes and 2772 edges (Fig 3).

### 3.2. Common-target network

**Fig 4:**
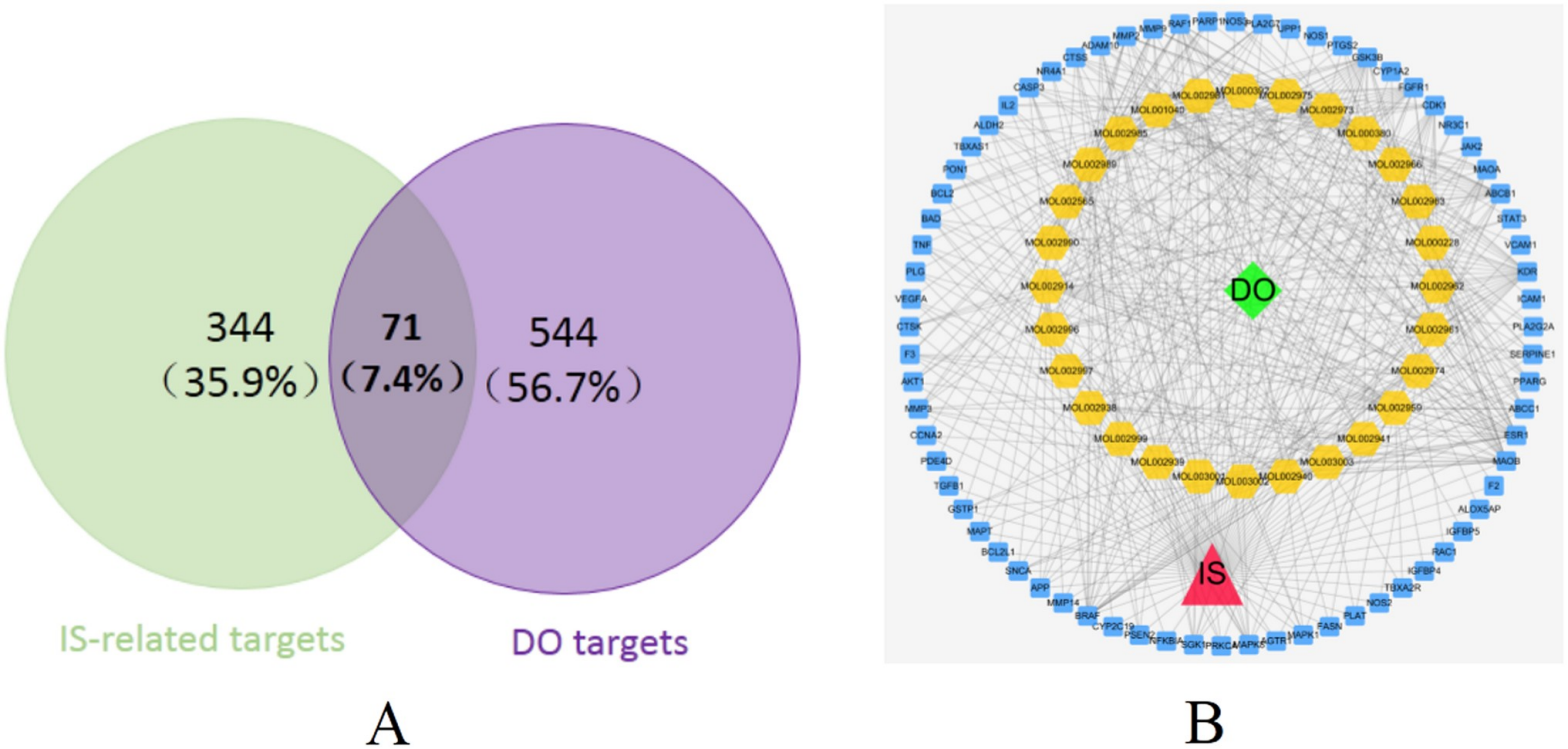
Common-targets network. (A) 71 targets are common to DO and IS. (B) Common-target network, including 101 nodes and 498 edges. Yellow nodes stand for bioactive components from DO, blue nodes stand for targets.

The occurrence and development of IS involves the co-regulation of multiple genes. We screened 126, 196, and 129 targets from 3 databases (NCBI, CTD, and GeneCards), and collected a total of 344 targets related to IS. Using Venny online drawing tool, 544 monomer component targets and 344 disease targets were intersected, and a total of 71 common targets were obtained (Fig 4A, Table 1). Then, we built a complex network based on the interactions among bioactive components, targets and the disease (IS) by using the Cytoscape software and gained a network which was made up of 101 nodes and 498 edges (Fig 4B). The importance of the node in terms of degree and intermediary degree was reflected using Cytoscape software for topological analysis on the obtained network graph. The values of nodes and betweenness centrality were commonly used to describe the importance of network nodes.

**Table 1:** 71 common targets among the targets related to DO and the targets related to IS.

Number	Uniprot ID	Target name	Target name abbreviation
1	P27338	Monoamine oxidase B	MAOB
2	P03372	Estrogen receptor alpha	ESR1
3	P33527	Multidrug resistance-associated protein 1	ABCC1
4	P37231	Peroxisome proliferator-activated receptor gamma	PPARG
5	P05121	Plasminogen activator inhibitor-1	SERPINE1
6	P14555	Phospholipase A2 group IIA	PLA2G2A
7	P35968	Vascular endothelial growth factor receptor 2	KDR
8	P40763	Signal transducer and activator of transcription 3	STAT3
9	P08183	ATP-dependent translocase ABCB1	ABCB1
10	P21397	Monoamine oxidase A	MAOA
11	O60674	Tyrosine-protein kinase JAK2	JAK2
12	P06493	Cyclin-dependent kinase 1	CDK1
13	P11362	Fibroblast growth factor receptor 1	FGFR1
14	P49841	Glycogen synthase kinase-3 beta	GSK3B
15	P35354	Prostaglandin G/H synthase 2	PTGS2
16	Q16831	Uridine phosphorylase 1 (by homology)	UPP1
17	Q13093	Platelet-activating factor acetylhydrolase	PLA2G7
18	P29474	Nitric-oxide synthase, endothelial (by homology)	NOS3
19	P37840	Alpha-synuclein	SNCA
20	P05067	Amyloid-beta precursor protein	APP
21	P13726	Tissue factor	F3
22	P10636	Microtubule-associated protein tau	MAPT
23	P35228	Nitric oxide synthase, inducible	NOS2
24	P05091	Aldehyde dehydrogenase2	ALDH2
25	P08254	Matrix metalloproteinase 3	MMP3
26	P04049	Serine/threonine-protein kinase RAF	RAF1
27	P15056	Serine/threonine-protein kinase B-raf	BRAF
28	P08253	Matrix metalloproteinase 2	MMP2
29	P63000	Ras-related C3 botulinum toxin substrate 1	RAC1
30	P20292	Arachidonate 5-lipoxygenase-activating protein	ALOX5AP
31	P00734	Prothrombin	F2
32	P50281	Matrix metalloproteinase 14	MMP14
33	P15692	Vascular endothelial growth factor A	VEGFA
34	P43235	Cathepsin K	CTSK
35	P31749	Serine/threonine-protein kinase AKT	AKT1
36	P10415	Apoptosis regulator Bcl-2	BCL2
37	P14780	Matrix metalloproteinase 9	MMP9
38	P09874	Poly [ADP-ribose] polymerase-1	PARP1
39	O14672	ADAM10	ADAM10
40	P25774	Cathepsin S	CTSS
41	P22736	Nuclear receptor subfamily 4 group A member 1	NR4A1
42	P42574	Caspase-3	CASP3
43	P60568	Interleukin-2	IL2
44	P24557	Thromboxane-A synthase	TBXAS1
45	P27169	Serum paraoxonase/arylesterase 1	PON1
46	Q92934	Bcl2-antagonist of cell death	BAD
47	P01375	TNF-alpha	TNF-α
48	P00747	Plasminogen	PLG
49	Q08499	Phosphodiesterase 4D	PDE4D
50	P09211	Glutathione S-transferase Pi	GSTP1
51	P33261	Cytochrome P450 2C19	CYP2C19
52	P49810	Presenilin-2	PSEN2
53	P25963	NF-kappa-B inhibitor alpha	NFKBIA
54	O00141	Serine/threonine-protein kinase Sgk1	SGK1
55	P17252	Protein kinase C alpha type	PRKCA
56	P45983	c-Jun N-terminal kinase 1	MAPK8
57	P30556	Type-1 angiotensin II receptor (by homology)	AGTR1
58	P28482	MAP kinase ERK2	MAPK1
59	P49327	Fatty acid synthase	FASN
60	P00750	Tissue-type plasminogen activator	PLAT
61	P21731	Thromboxane A2 receptor	TBXA2R
62	P22692	Insulin-like growth factor binding protein 4	IGFBP4
63	P24593	Insulin-like growth factor binding protein 5	IGFBP5
64	P05362	Intercellular adhesion molecule-1	ICAM1
65	P19320	Vascular cell adhesion protein 1	VCAM1
66	P04150	Glucocorticoid receptor	NR3C1
67	P05177	Cytochrome P450 1A2	CYP1A2
68	P29475	Nitric-oxide synthase, brain	NOS1
69	P01137	Transforming growth factor beta-1	TGFB1
70	Q07817	Apoptosis regulator Bcl-X	BCL2L1
71	P20248	CDK2/Cyclin A	CCNA2

### 3.3. PPI network construction

**Fig 5:**
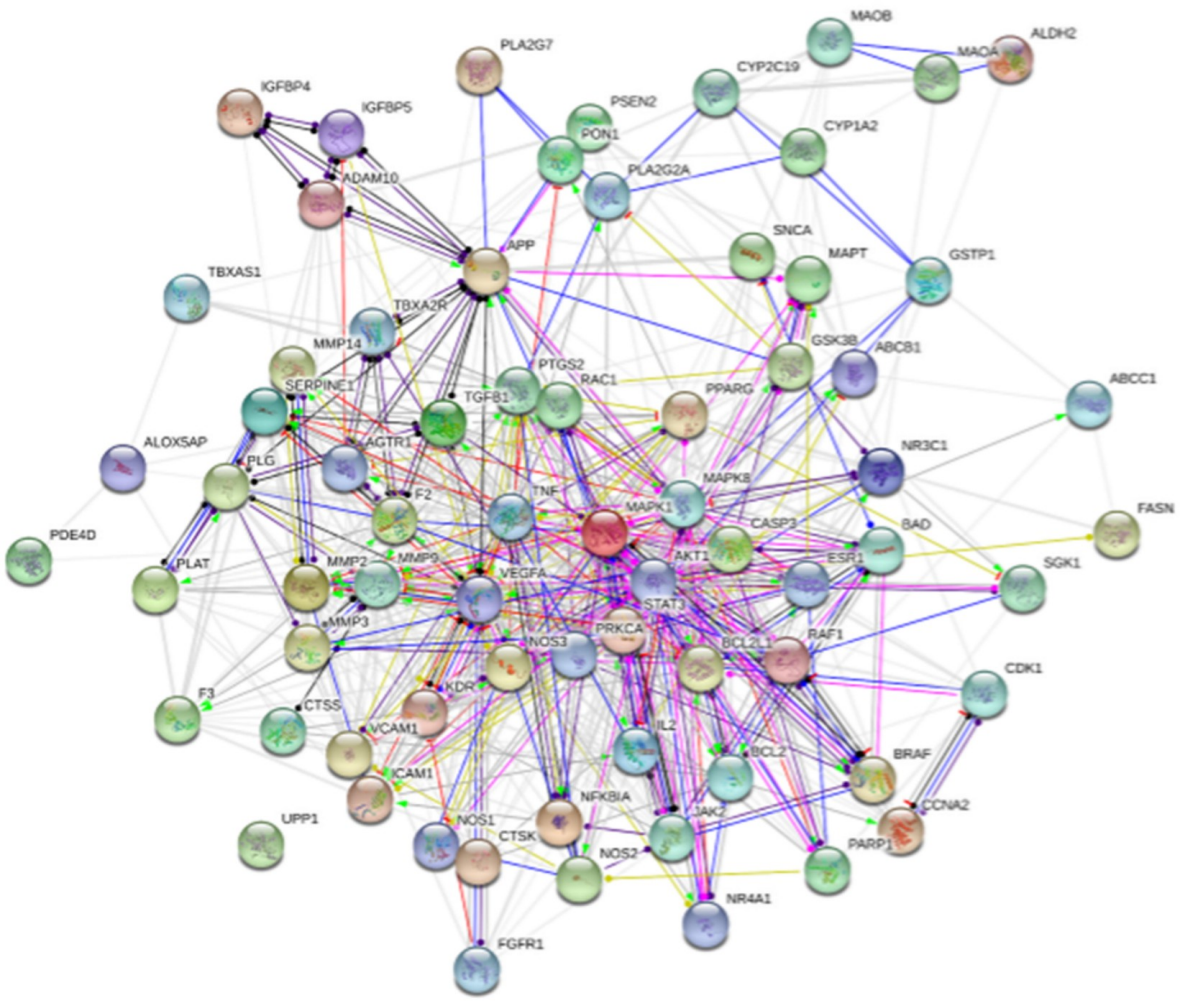
Protein–protein interaction (PPI) networks of bioactive components of DO for the treatment of IS. Each node represents the relevant gene, the edge means line thickness indicates the strength of data support.

In this study, we used the string tool to acquire PPI network for the 71 overlapped targets. With a combined score greater than 0.4 and ‘Homo sapines’as selecting criterions, the network of PPI consisted of 71 nodes and 815 edges (Fig 5). Each node represents the relevant gene, the edge means line thickness indicates the strength of data support.

### 3.4. Core target network

**Fig 6:**
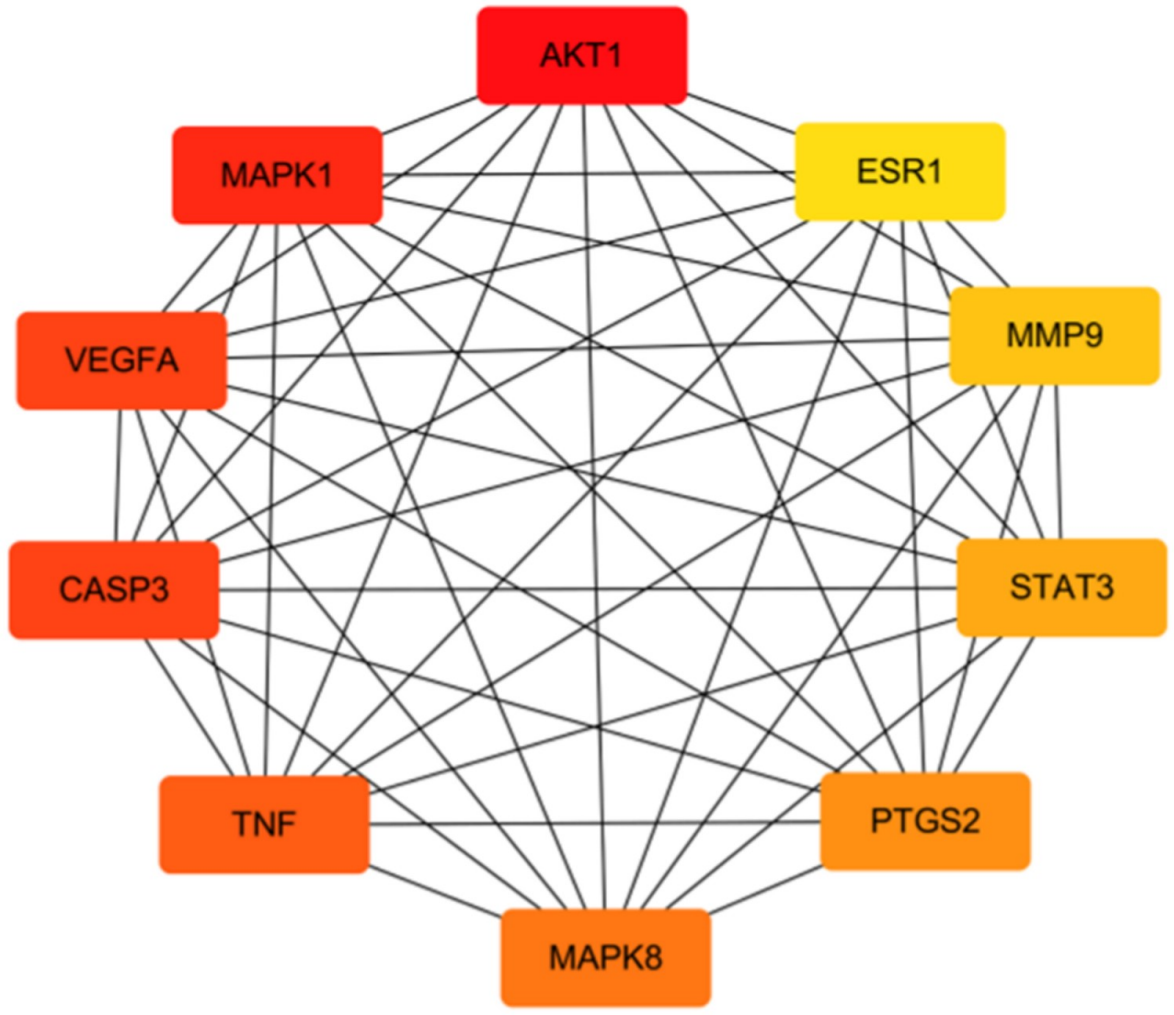
Hub top 10 genes in PPI network, the darker the color, the higher the score.

Top10 core targets in the PPI network were obtained by cytoHubba plug-ins, followed by AKT1, MAPK1, VEGFA, CASP3, TNF, MAPK8, PTGS2, STAT3, MMP9 and ESR1 (Fig 6). The darker the node color, the more targets are connected to it and the greater its importance is of the possible role in the occurrence and development of IS.

### 3.5. Screening of potential bioactive components

**Fig 7:**
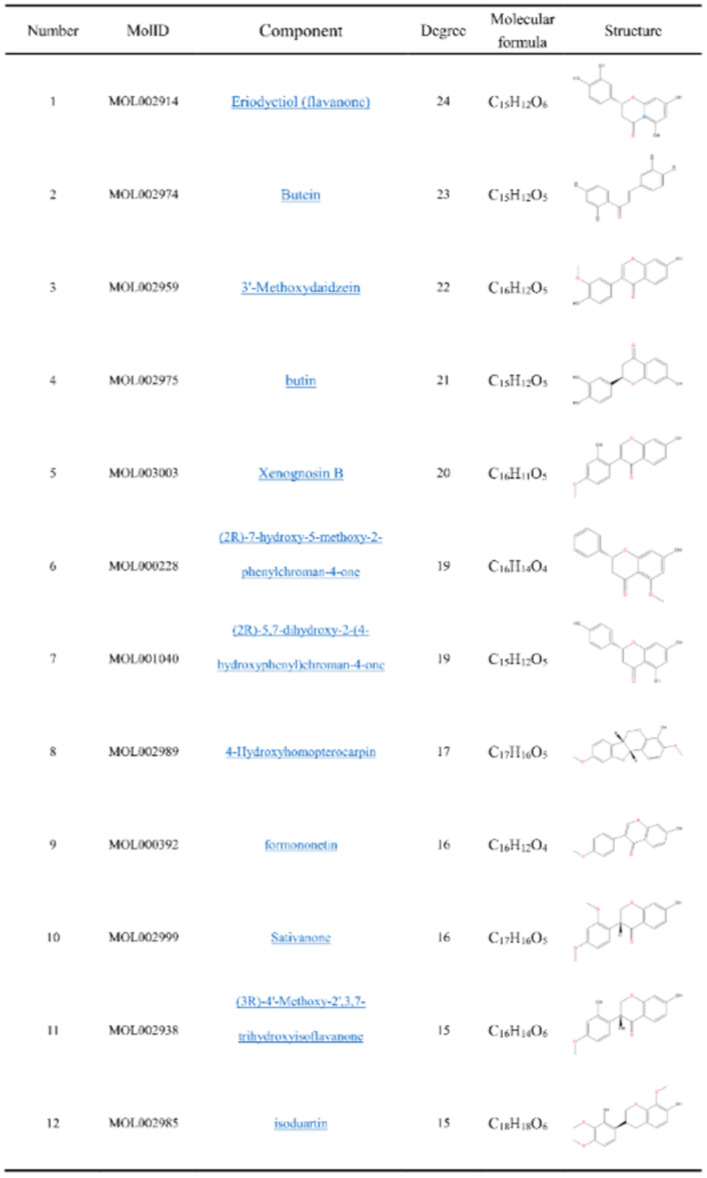
Potential bioactive components of DO treating IS.

We screened 12 potential bioactive components using the ‘Degree>median’ and ‘Degree>14’ as screening criteria (Fig 7).

We analyzed the structure of 12 compounds screened by network pharmacology and found that these were flavonoids, including dihydroflavonoids (1. MOL002914, 4. MOL002975, 6. MOL000228, 7. MOL001040, 8. MOL002989, 10. MOL002999, 11. MOL002938, 12. MOL002985), isoflavones (2. MOL002974, 3. MOL002959, 5. MOL003003, 9. MOL000392). Studies have shown that the flavonoids of DO have anti-inflammatory, anti-oxidant, anti-thrombotic, anti-platelet aggregation, anti-tumor and other pharmacological effects.

### 3.6. Enrichment analysis

GO functional enrichment analysis and KEGG pathway enrichment in DAVID resulted in 182 entries (*P*<0.05) and 107 signaling pathways (*P*<0.05). Among them, GO functional enrichment analysis included 148 biological processes (BPs), 16 cell compositions (CCs), and 18 molecular functions (MFs), accounting for 81%, 9%, and 10%, respectively. The biological processes mainly involved lipopolysaccharide-mediated signaling pathway, positive regulation of protein phosphorylation, cell response to organic cyclic compound, cellular response to vascular endothelial growth factor stimulus, angiogenesis, apoptotic process, positive regulation of nitric oxide biosynthesis process, response to drug and response to hypoxia. The cell compositions mainly involved extracellular space, platelet alpha granule lumen, extracellular region, proteinaceous extracellular matrix, plasma membrane and cell surface. The molecular functions mainly involved protein binding, enzyme binding, serine-type endopeptidase activity, metallopeptidase activity and protease binding.

According to IS pathogenesis, these biological processes can be divided into 3 parts, including oxidative stress (GO:0071407, GO:0032355, GO:0045429, GO:0051926, GO:0070374, GO:0043066, KEGG:04151, KEGG:04068, KEGG:04915, KEGG:04010, KEGG:04014), inflammatory response (GO:0043066, GO:0071222, GO:0071260, KEGG:04668, KEGG:04010, KEGG:04014, KEGG:04064) and regulation of vascular endothelial function (GO:0043536, GO: 0045766, GO:0001666, GO:0071456, GO:0043066, KEGG:04370, KEGG: 04066) (Table 2).

**Table 2:** Functions of 71 common targets based on GO and KEGG pathway analysis through DAVID and ClueGO.

Classification	ID	Term
Oxidative stress	GO:0071407	cellular response to organic cyclic compound
	GO:0032355	response to estradiol
	GO:0045429	positive regulation of nitric oxide biosynthetic process
	GO:0051926	negative regulation of calcium ion transport
	GO:0070374	positive regulation of ERK1 and ERK2 cascade
	GO:0043066	negative regulation of apoptotic process
	KEGG:04151	PI3K-Akt signaling pathway
	KEGG:04068	FoxO signaling pathway
	KEGG:04915	Estrogen signaling pathway
	KEGG:04010	MAPK signaling pathway
	KEGG:04014	Ras signaling pathway
Inflammatory response	GO:0031663	Lipopolysaccharide-mediated signaling pathway
	GO: 0071222	cellular response to lipopolysaccharide
	GO:0071260	Cellular response to mechanical stimulus
	KEGG:04668	TNF signaling pathway
	KEGG:04064	NF-κB signaling pathway
	KEGG:04010	MAPK signaling pathway
	KEGG:04014	Ras signaling pathway
Vascular endothelial function regulation	GO: 0043536	positive regulation of blood vessel endothelial cell migration
	GO: 0045766	positive regulation of angiogenesis
	GO:0001666	Response to hypoxia
	GO:0071456	Cellular response to hypoxia
	GO:0043066	Negative regulation of apoptotic process
	KEGG:04066	HIF-1 signaling pathway
	KEGG:04370	VEGF signaling pathway

### 3.7. Molecular docking

**Fig 8:**
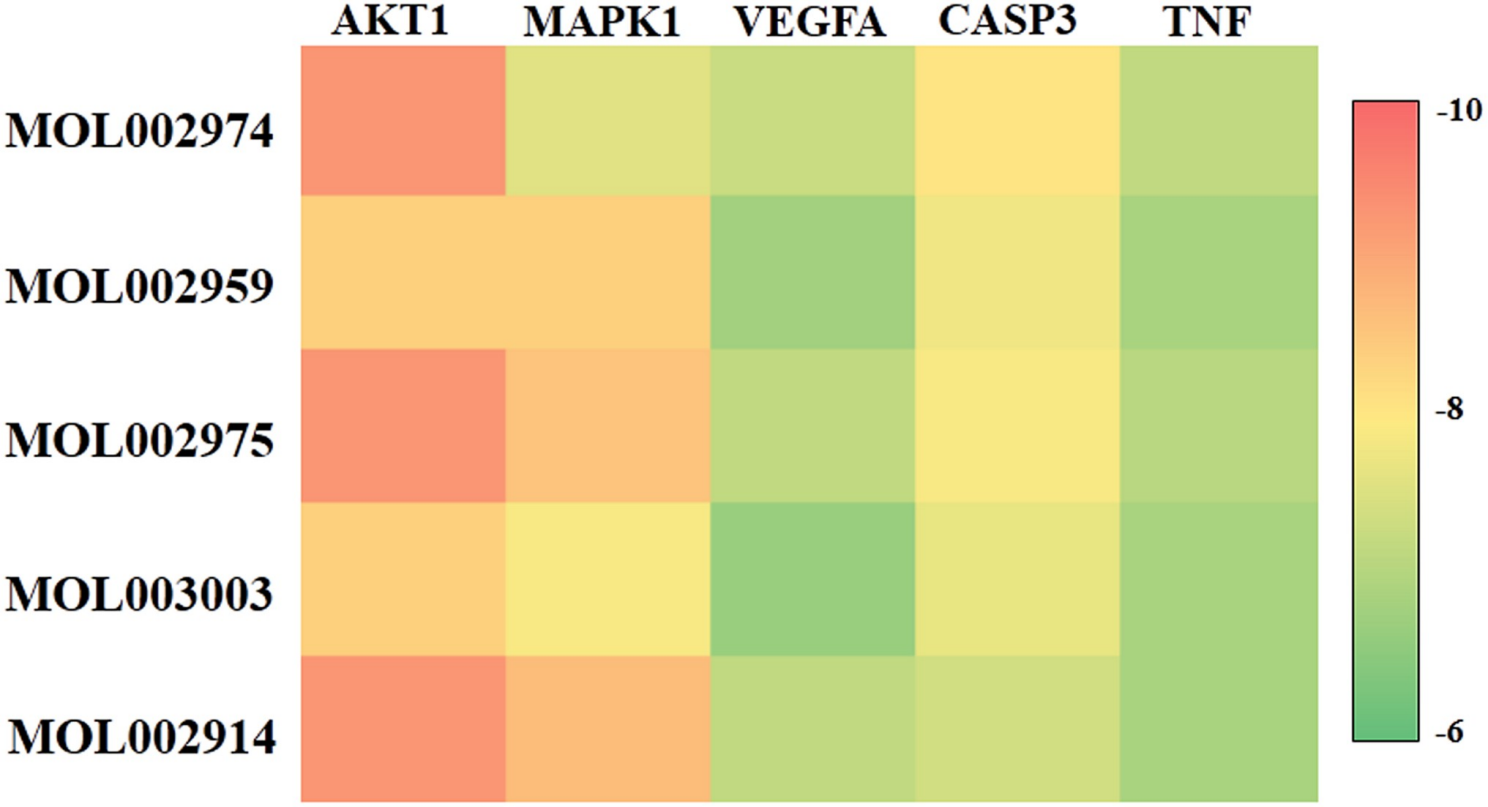
Heat map of the difference of binding energy between potential bioactive components and top5 core targets.

**Fig 9:**
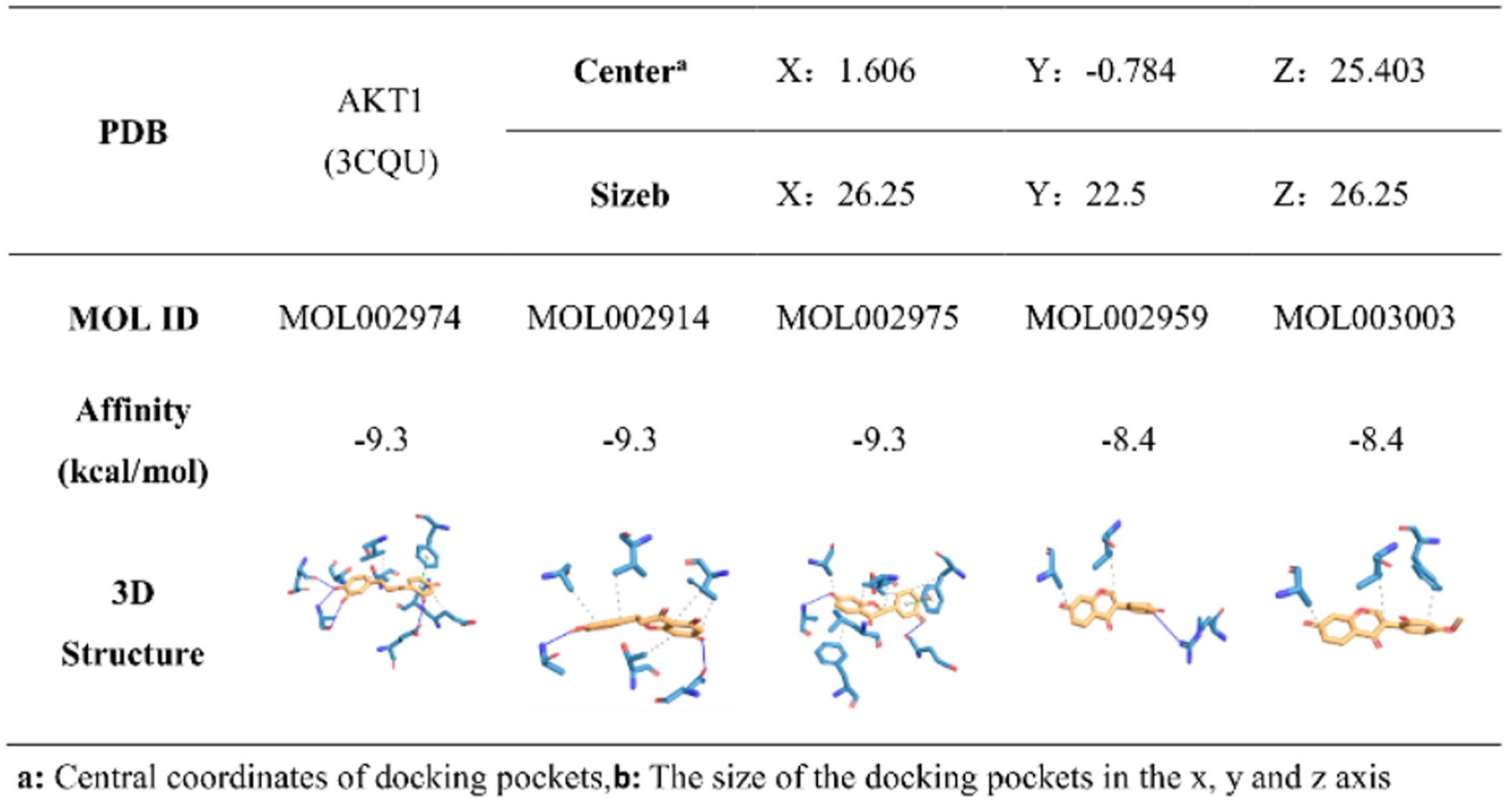
Optimal docking of potential bioactive components with top5 core targets.

**Fig 10:**
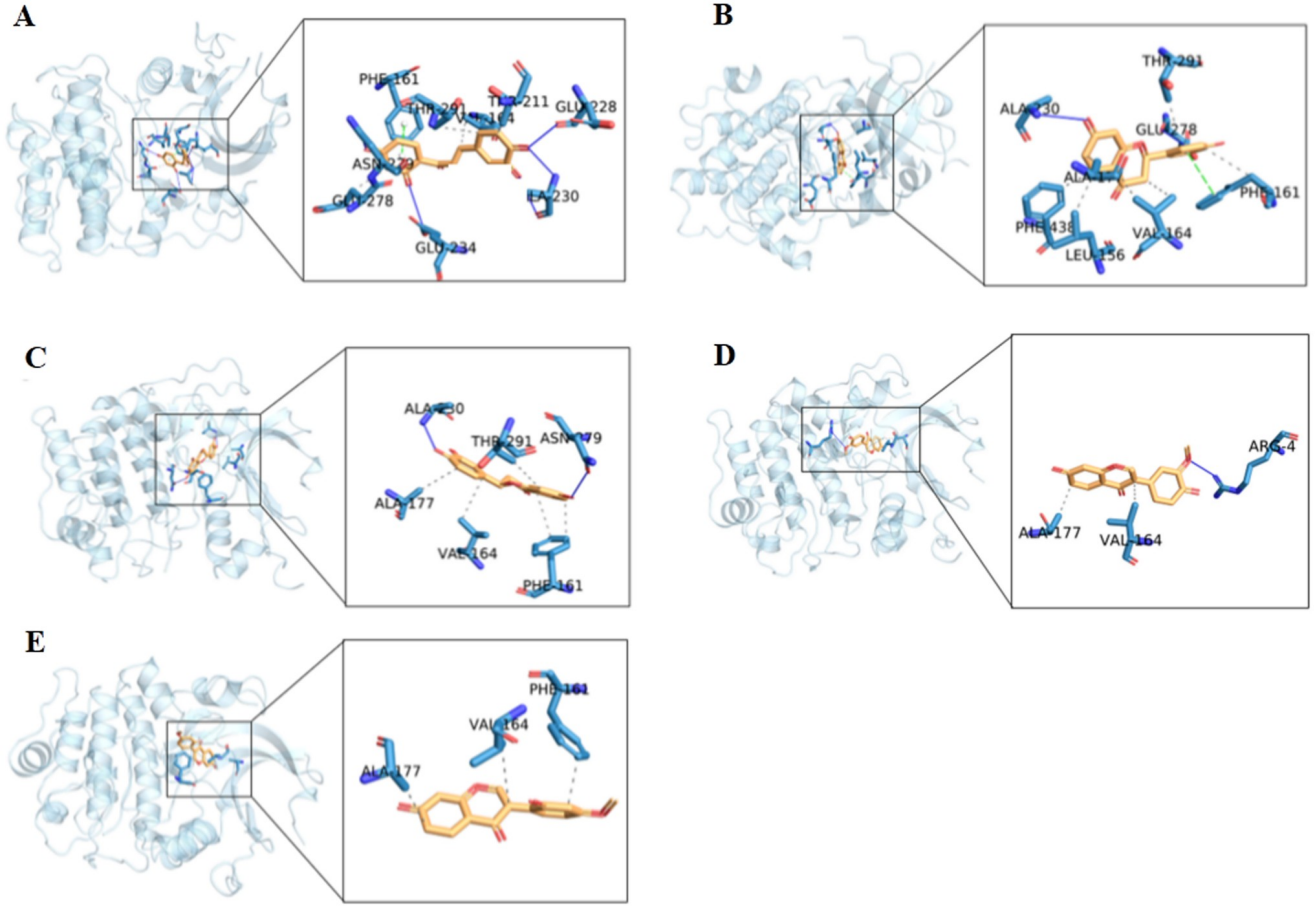
The binding site of the potential bioactive components and the core target protein AKT1. A:MOL002974, B:MOL002975, C:MOL002914, D:MOL002959, E:MOL003003. The blue solid lines represented hydrogen bondings, and the gray dashed lines represented hydrophobic interactions.

Molecular docking is one of the most important and commonly used methods for comparing the biological activity of molecules on enzymes, and the most important parameter of molecular docking is affinity. The molecule with the lowest value of this parameter has the highest biological activity. To further screened the core bioactive components of DO affecting on IS, we tested the affinity of 5 potential bioactive components and the following top5 core target proteins: AKT1 (PDB: 3CQU), MAPK1 (PDB: 5K4I), VEGFA (PDB: 3BDY), CASP3 (PDB: 3H0E) and TNF (PDB: 4TWT), respectively (Fig 8). The results showed that all the 5 potential bioactive components could stably bind to the top5 targets and strongly bind to the target protein AKT1, so we focused on analyzing the binding sites of MOL002974, MOL002914 and MOL002975 with AKT1 (Fig 9). According to the ligand-protein interaction after molecular docking, it could be found that the binding of each small molecule to the target protein mainly depended on hydrophobic interaction and hydrogen bonding (Table 3). MOL002974 combined with residues such as THR211, THR291, GLU278, PHE161, VAL164 on the AKT1 binding site to form a hydrophobic interaction, and combined with the residues of ASN279, GLU228, ALA230 and GLU234 to form hydrogen bonds (Fig 10A). MOL002975 combined with the residues of THR291, PHE161, ALA177, VAL164 and LEU156 on the AKT1 binding site to form a hydrophobic interaction, and combined with the residues of ALA230 and GLU278 to form hydrogen bonds (Fig 10B). MOL002914 combined with the residues such as THE291, ALA177, VAL164, PHE161 on the AKT1 binding site to form a hydrophobic interaction, and combined with the residues of ALA230 and ASN279 to form hydrogen bonds (Fig 10C). MOL2959 binded with the residues of ALA177 and VAL164 on the AKT1 binding site to form a hydrophobic interaction, and combined with the residues of ARG4 to form hydrogen bonds (Fig 10D). MOL003003 binded with the residues of ALA177, VAL164 and PHE161 on the AKT1 binding site to form a hydrophobic interaction (Fig 10E).

**Table 3:** The binding site of the potential bioactive components and the core target protein AKT1.

Location	MOL002974	MOL002914	MOL002975	MOL002959	MOL003003
THR211	**√**				
THR291	**√**	**√**	**√**		
ASN279	**√**	**√**			
GLU278	**√**		**√**		
PHE161	**√**	**√**	**√**		**√**
VAL164	**√**	**√**	**√**	**√**	**√**
GLU228	**√**				
ALA230	**√**	**√**	**√**		
GLU234	**√**				
ALA177		**√**	**√**	**√**	**√**
LEU156			**√**		
PHE438			**√**		
ARG4				**√**	
Number	9	6	8	3	3

### 3.8. Experimental verification

#### 3.8.1. Cell viability analysis

**Fig 11:**
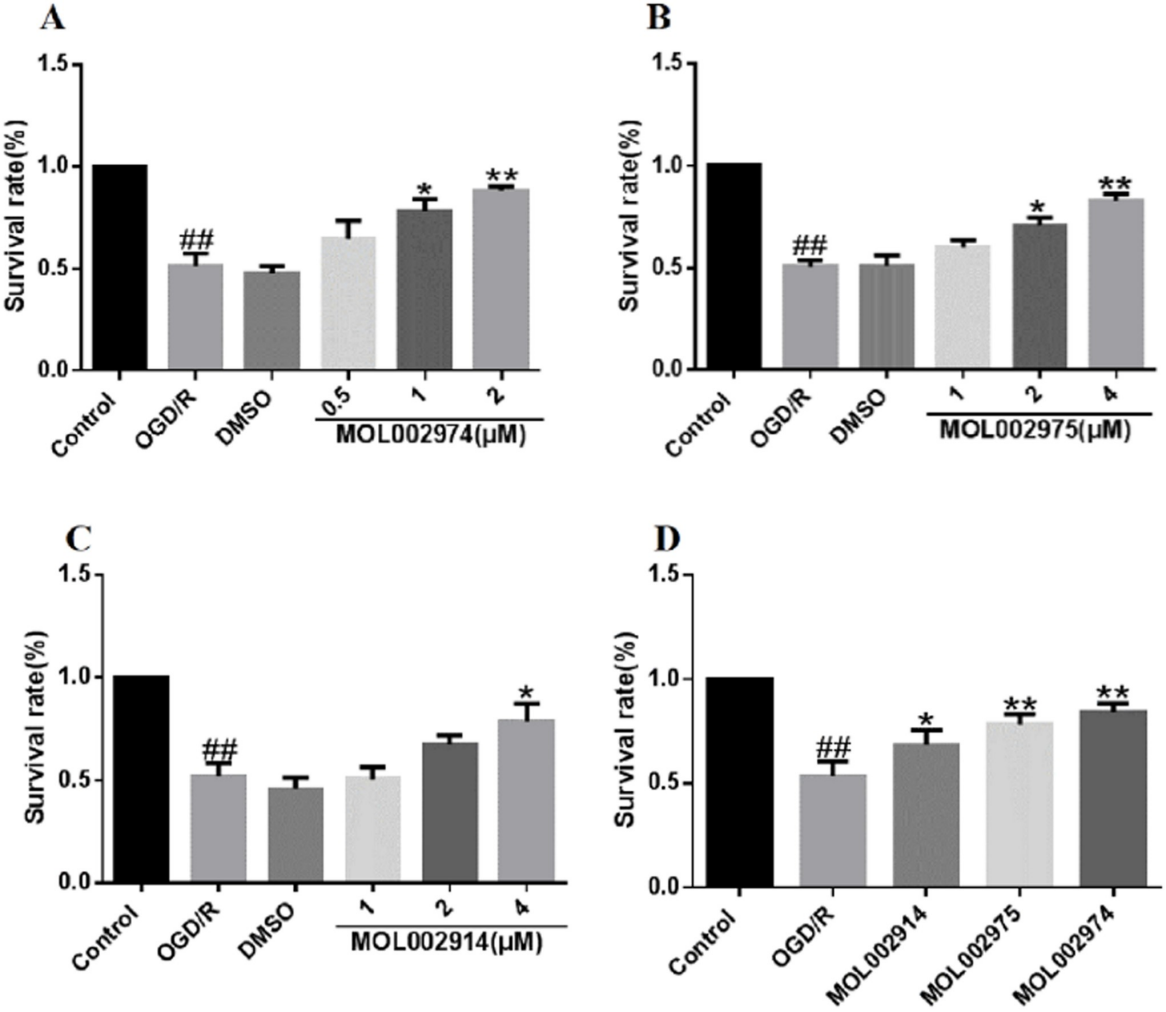
Effects of 3 core bioactive components on the survival rate of PC12 cells. A: MOL002974, B: MOL002975, C: MOL002914, D: 3 potential bioactive components. ^*##*^ *P*<0.01 vs control group; *P<0.05, **P<0.01 vs OGD/R group. ($\overset{-}{X}\pm SD$, n = 3).

Compared with the Control group, the cell survival rate of the OGD/R group was significantly reduced (*P*<0.01), suggesting that OGD/R caused PC12 cell damage. Compared with the OGD/R group, the MOL002974 low-dose groups and MOL002975 low-dose groups had a tendency to increase cell survival, but there was no significant difference. The survival rate of cells in the medium-dose group (*P*<0.05) and high-dose group (*P*<0.01) gradually increased (Fig 11A and 11B). MOL002914 low-dose group and medium-dose group had a tendency to increase cell survival rate, but there was no significant difference. The high-dose group (*P*<0.05) increased cell survival rates (Fig 11C). It is suggested that MOL002974, MOL002975, and MOL002914 could promote PC12 cells survived in a dose-dependent manner. The effects of the 3 core bioactive components on the survival rate of PC12 cells were further compared at the same concentration, and the results showed that MOL002974 had a slightly better effect on improving the survival rate of cells (Fig 11D).

#### 3.8.2. Western blot analysis

**Fig 12:**
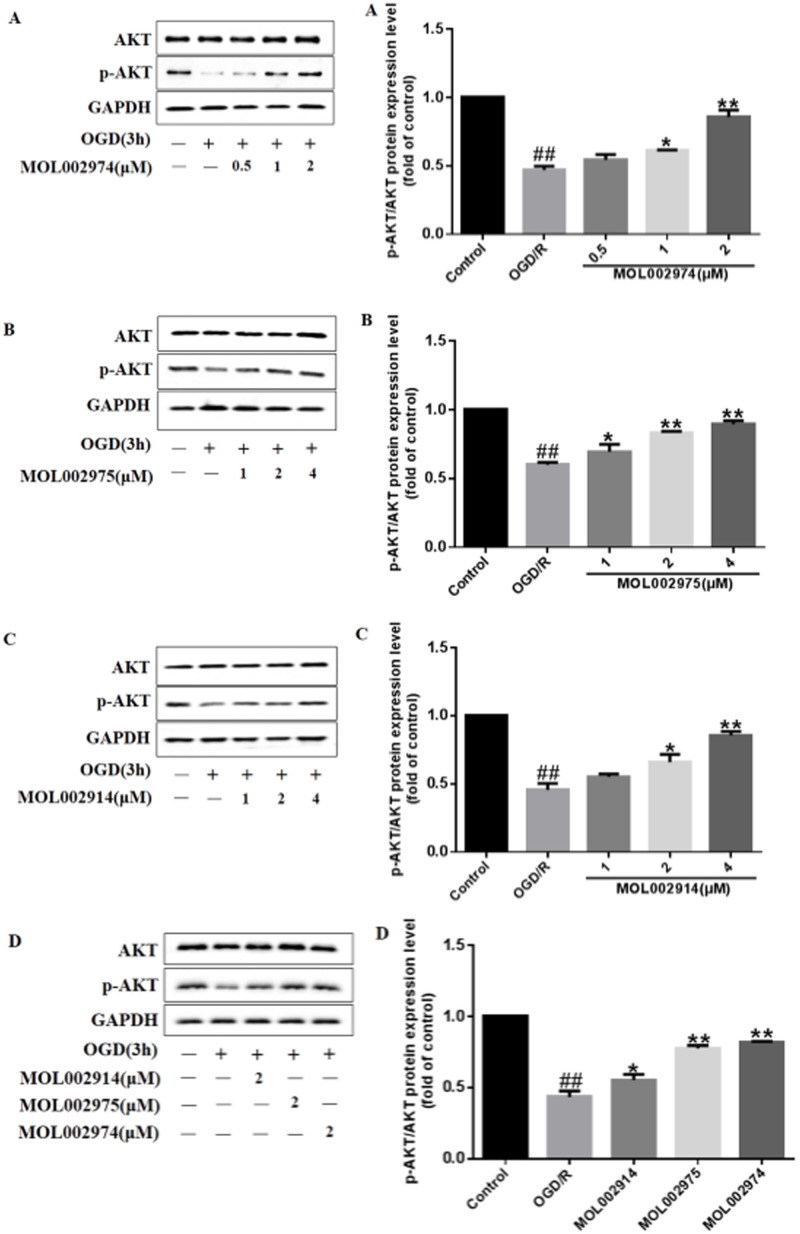
Effects of core bioactive components on the core target protein expression of p-AKT/AKT induced by OGD/R in PC12 cells. A: MOL002974, B: MOL002975, C: MOL002914, D: 3 potential bioactive components. Values are represented as mean ± SD from 3 independent experiments, each experiment repeated 3 times^.#^*P*<0.05 vs. Control group,^##^*P*<0.01 vs. Control group,*P<0.05 vs. OGD/R group, **P<0.01 vs. OGD/R group.

Based on the results of network pharmacology and molecular docking, we used western blotting to verify the regulation of the core bioactive components MOL002974 (Fig 12A), MOL002975 (Fig 12B) and MOL002914 (Fig 12C) on the target of AKT in PC12 cells. The results showed that, compared with the control group (no treatment), the OGD/R group inhibited the phosphorylation of AKT (p<0.01). However, 3 core bioactive component treatments significantly up-regulated the phosphorylation of AKT in a dose dependent manner (p<0.01), respectively. In addition, 3 core bioactive components could up-regulate AKT phosphorylation under the same conditions, and MOL002974 had a slightly better effect (Fig 12D).

#### 3.8.3. Neurofunctional scores analysis

**Fig 13:**
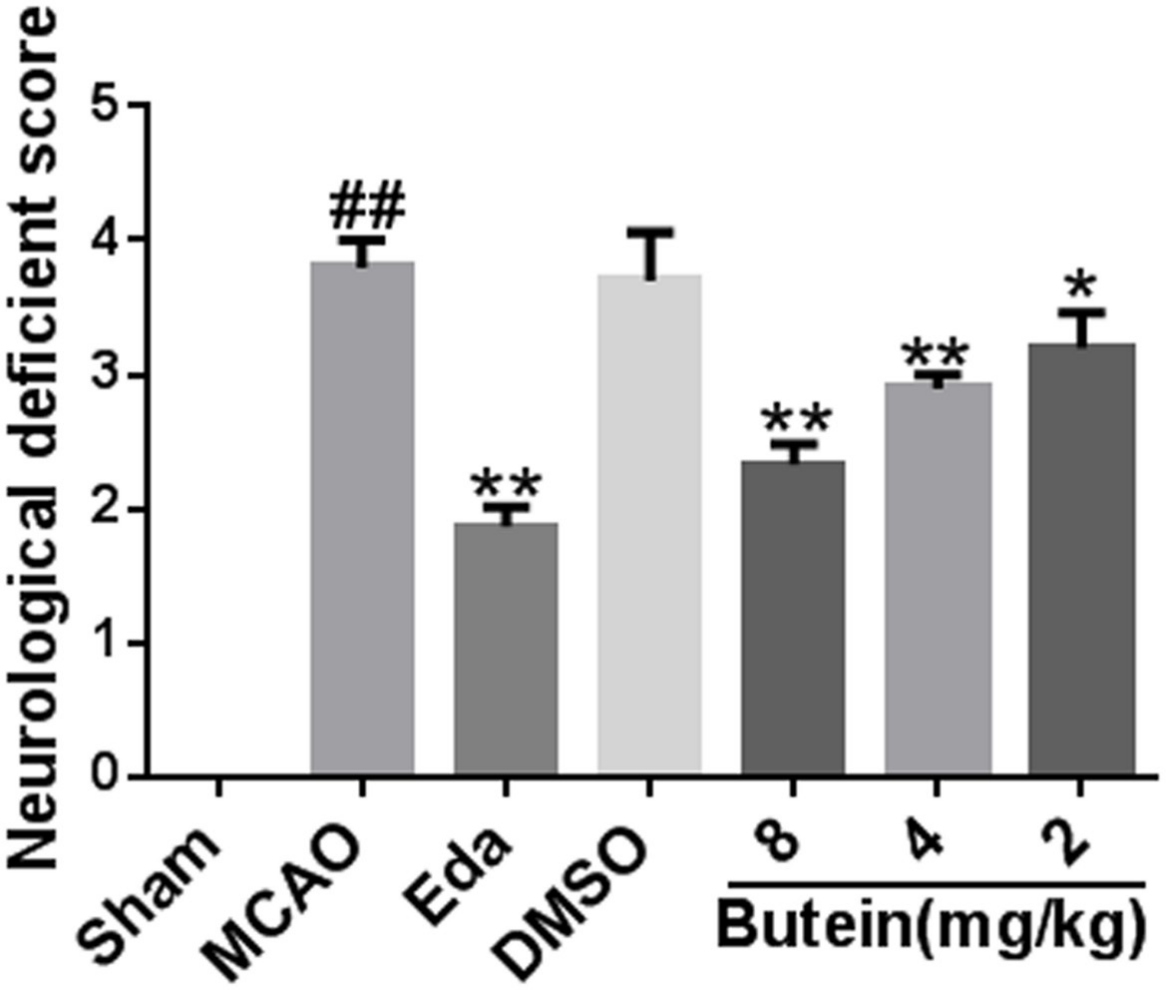
Effects of butein on neurofunctional scores in MCAO rats. ^*##*^*P*<0.01 vs Sham group; *P<0.05, **P<0.01 vs MCAO group. ($\overset{-}{X}\pm SD$, n = 6).

The rats in each group were scored for neurological function before being sacrificed. Compared with the Sham group, the neurofunctional scores of rats in the MCAO group increased significantly (*P*<0.01). Compared with the MCAO group, butein low-dose (*P*<0.05), medium-dose (*P*<0.01), high-dose group (*P*<0.01) and Eda group (*P*<0.01) significantly reduced the neurological scores of rats, suggesting butein can reduce cerebral damage caused by CI/R in a dose-dependent manner (Fig 13).

#### 3.8.4. Cerebral infarct volume analysis

**Fig 14:**
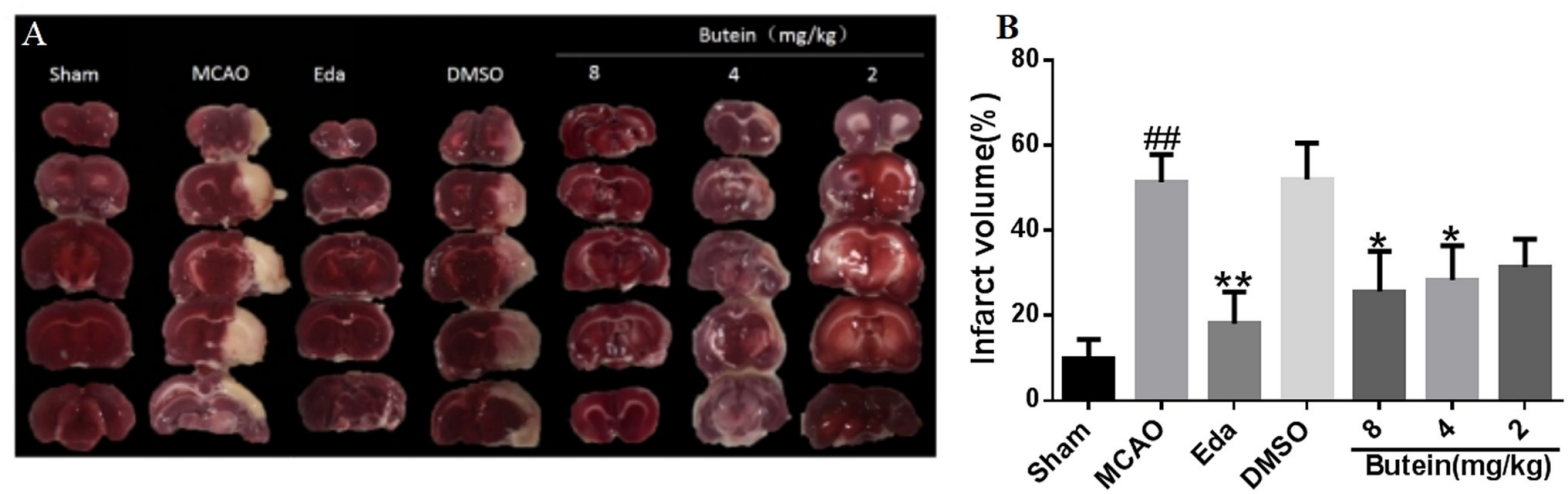
Effects of butein on cerebral infarct volume of MCAO rat. A: TTC stained cerebral sections of each group; B: Percentage of TTC stained infarct volume. ^*##*^ *P*<0.01 vs Sham group; *P<0.05, **P<0.01 vs MCAO group. ($\overset{-}{X}\pm SD$, n = 3).

Compared with the Sham group, the cerebral infarction volume of rats in the MCAO group increased significantly (*P*<0.01). Compared with the MCAO group, butein medium-dose (*P*<0.05), high-dose group (*P*<0.05) and Eda group (*P*<0.01) significantly reduced cerebral infarct volume in rats, suggesting butein can reduce cerebral damage caused by CI/R in a dose-dependent manner (Fig 14).

#### 3.8.5. LDH levels analysis

**Fig 15:**
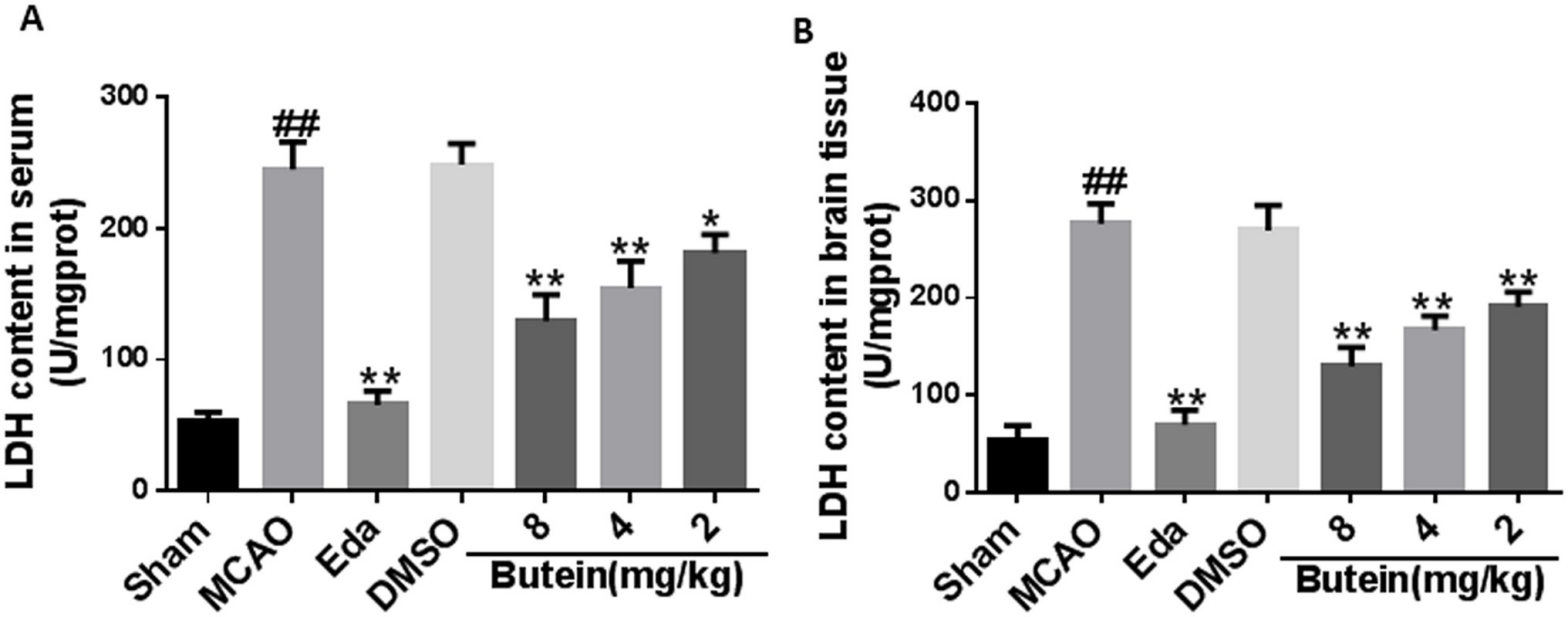
Effects of butein on LDH levels in MCAO rats. A: Effects of butein on LDH level in serum of MCAO rats. B: Effects of butein on cerebral tissue LDH level of MCAO rats. ^*##*^ *P*<0.01 vs Sham group; *P<0.05, **P<0.01 vs MCAO group. ($\overset{-}{X}\pm SD$, n = 3).

Compared with the Sham group, LDH levels in the cerebral tissue and serum of rats in MCAO group was significantly increased (*P*<0.01), suggesting that MCAO caused cerebral damage. Compared with the MCAO group, butein low-dose (*P*<0.05, *P*<0.01), medium-dose (*P*<0.01), high-dose (*P*<0.01) and the Eda group (*P*<0.01) significantly reduced the LDH levels, suggesting that butein could inhibit the release of LDH, and showed a dose-dependent manner (Fig 15).

## 4. Discussion

IS is a major threaten to the human, however, there was no effective drugs used in clinical besides thrombolytic drugs. There are multiple biological processes in IS, including oxidative stress, inflammation, apoptosis, and energy metabolism disorders. A single drug may difficult to achieve therapeutic effects, drug cocktail therapy or TCM containing multiple compounds may be an effective treatment method.

DO has good effects in regulating qi and blood circulation, and is commonly used for treating IS. DO contains a large number of bioactive components, which are mainly flavonoids, including dihydroflavonoids, chalcone, isoflavones and so on. However, the effective substances and active mechanisms for the treatment of IS are still unclear. In this study, we conducted component screening, target prediction and network analysis through network pharmacology to study the pharmacological mechanism related to DO and IS, which improved the accuracy of target prediction to a certain extent. Based on the analysis of the PPI system, it was found that 10 highly differentially expressed genes such as AKT1, MAPK1, VEGFA, CASP3, TNF, MAPK8, PTGS2, STAT3, MMP9 and ESR1 played a key role in the pharmacological function of DO and were considered to be core targets. Network topology analysis showed that the process of DO affecting IS involved a variety of biological processes, cell composition and molecular functions, and was a complex process. At the same time, the core targets were significantly enriched in PI3K/Akt signaling pathway, TNF signaling pathway, MAPK signaling pathway, NF-κB signaling pathway, FoxO signaling pathway, Ras signaling pathway, Estrogen signaling pathway and other signaling pathways.

IS involved a variety of biological processes, including oxidative stress, inflammation and vascular endothelial regulation. Among them, oxidative stress was an important pathological link of nerve function injury under ischemia and hypoxia [21, 22], which was closely related to PI3K/Akt signaling pathway, FoxO signaling pathway, Estrogen signaling pathway, Ras signaling pathway and other signaling pathways. PI3K/Akt was a classical signal transduction pathway that regulated cell survival, differentiation and apoptosis, and played an important biological role by regulating the downstream apoptosis-related proteins. A large number of studies had found that the PI3K/Akt pathway was a pro-survival signaling pathway, and the activation of the pathway helped to play a protective role on nerve cells, especially when the ischemic/hypoxic neurons were damaged [23–25]. The activated Akt could initiate the downstream cascade reaction of the PI3K/Akt signaling pathway, further phosphorylate a series of substrates, such as downstream Bad, Caspase-3, GSK-3β and exert its function of regulating cell differentiation, promoting cell survival and anti-apoptosis through various channels [26]. Studies had shown that [27] activating the PI3K/Akt signaling pathway could inhibit the activation of Caspase-3 to play a central neuroprotective effect. In addition, studies had found that [28] estradiol could increase cell viability, reduce the production of Reactive oxygen species (ROS), activate Akt signal and inhibit GSK-3β involved in neurodegenerative changes, promote the separation of Nrf2 from Keap1, and significantly increase HO-1 expression and SOD activity. The inflammatory response ran through the whole process of the occurrence and development of IS.

After cerebral ischemia, extensive brain tissue necrosis occurred in the ischemic region because of energy depletion, which released a large number of inflammatory mediators, thus activating the immune response and further promoting the release of inflammatory factors [29]. It was closely related to TNF signaling pathway, NF-κB signaling pathway, MAPK signaling pathway and other signaling pathways. MAPK was an important transmitter of signals from the cell surface to the inside of the nucleus. They regulated many physiological activities, such as inflammation and apoptosis [30]. MAPK activated pro-inflammatory factors such as TNF-α, interleukin family (IL1, IL6, etc.) to exacerbate the inflammatory response [31]. TNF-α was a pro-inflammatory factor with multiple pro-inflammatory and neurotoxic effects. It was the initiating factor of inflammatory response and had complex biological activities. During the early stage of cerebral ischemia, increased TNF-α secretion or synthesis was the main cause of cerebral infarction [32]. Experiments had shown that an increase in the amount of TNF-α could promote the inflammatory response after cerebral ischemia/reperfusion (CI/R) and aggravated brain damage [33], while TNF-α inhibitors could reduce CI/R injury [34]. In addition, studies had shown that inhibiting the expression of NF-κB could reduce the cerebral infarction area and neuronal death in MCAO rats [35]. The function of vascular endothelial regulation was closely related to VEGF signaling pathway and HIF-1 signaling pathway. HIF-1α was a hypoxia-induced nuclear transcription factor. Activated HIF-1α under ischemia-hypoxia state was activated, induced the transcriptional expression of downstream gene VEGF, participated in angiogenesis, and regulated cell adaptation to hypoxia [36].

Moreover, 5 potential bioactive components with degree≥20 among the 12 potential bioactive components were selected to be docked with the top5 core targets using Autodock Vina software to further analyze the possible core bioactive components that affected the IS according to the relationship between the potential components and the core target proteins. The results showed that the 5 potential bioactive components could bind stably with the top5 core targets which compared with the positive drugs, and the binding energy is lower, and the binding is more stable. It showed that in the process of DO affecting IS, these 5 potential bioactive components played an important role by regulating top5 core targets. Meanwhile, the 5 potential bioactive components showed better binding to AKT1 target, followed by MAPK1, CASP3, TNF and VEGFA. Based on the docking results, we focused on analyzing the binding sites of MOL002974, MOL002914 and MOL002975 with AKT1. In addition, the CCK8 method was used to detect the effects of 3 core bioactive components on the cell viability of PC12 cells. Western blot experiments were used to verify the regulatory effects of the 3 core bioactive components on the AKT of PC12 cells.

The cell viability results showed that MOL002974, MOL002975, and MOL002914 all improved the cell survival rate in a dose-dependent manner and alleviated the damage to PC12 cells in the OGD/R group, suggesting that the 3 core bioactive components could all promote cell survival. In addition, MOL002974 had the best effect on improving cell survival when the 3 core bioactive components are at the same concentration, suggesting that MOL002974 played a more important role in improving cell survival than the other 2 core bioactive components.

The results showed that, compared with the control group (no treatment), the OGD/R group inhibited the phosphorylation of AKT (p<0.01). However, 3 core bioactive component treatments significantly up-regulated the phosphorylation of AKT in a dose dependent manner (p<0.01), respectively. In addition, 3 core bioactive components could up-regulate AKT phosphorylation under the same conditions, and MOL002974 had a slightly better effect.

The results of *in vitro* experiments showed that OGD/R could inhibit cell survival and AKT phosphorylation which were reversed by the 3 core bioactive components. Among them, MOL002974 (butein) had a slightly better effect. Therefore, the protective effect of MOL002974 (butein) against cerebral ischemia was further evaluated in a rat model of middle cerebral artery occlusion (MCAO) by detecting neurological score, cerebral infarction volume and lactate dehydrogenase (LDH) level. The results indicated that MOL002974 (butein) could significantly improve the neurological score of rats, decrease cerebral infarction volume, and inhibit the level of LDH in the cerebral tissue and serum in a dose-dependent manner.

The binding sites of MOL002974, MOL002975, MOL002914 and the target protein AKT1 were 9, 8, and 6, respectively. Some of the binding sites were consistent, which may be the reason for the similar docking results of the 3 core bioactive components. Akt called protein kinase B (PKB), was a serine/threonine kinase that could be activated by catalyzing the phosphorylation of its own serine and threonine sites. MOL002974, MOL002975, MOL002914 and AKT1 binding sites had 2, 1, and 0 threonine residues, respectively. Therefore, we assumed that the more threonine residues in the binding site of AKT1, the bioactive components activation effect were better. Further experimental results showed that MOL002974 had the best effect on up-regulating AKT phosphorylation, which was in preliminary agreement with our hypothesis. The specific mechanism of action needs further experimental study and verification.

## 5. Conclusions

In summary, the results indicated that the bioactive components of DO may affect IS through important signaling pathways in the biological process of "oxidative stress", "inflammatory response" and "vascular endothelial function regulation", such as PI3K/Akt signaling pathway, TNF signaling pathway, MAPK signaling pathway. And through PPI network analysis, it was determined that 10 core targets including AKT1, MAPK1, VEGFA, CASP3, TNF, MAPK8, PTGS2, STAT3, MMP9 and ESR1, participated in these processes. The 5 potential bioactive components were docked with the top5 core targets by molecular docking software, and the 3 core bioactive components affecting the IS were further analyzed and verified by experiments. To a certain extent, this research reveals the potential mechanism of DO affecting IS, and provides a basis for the secondary development of DO.

## Supporting information

S1 Raw images (RAR)

S1 Data (RAR)
