# Supplementary material for: Network pharmacology and molecular docking reveal the effective substances and active mechanisms of Dalbergia Odoriferain protecting against ischemic stroke
Source: PLoS One. 2021 Sep 28;16(9):e0255736. doi: 10.1371/journal.pone.0255736 (PMC8478192; doi:10.1371/journal.pone.0255736)
Supplement: Attachment — Submitted filename: Response to Reviewers.docx [file pone.0255736.s003.docx]

**Dear editor**

Thank you for your letter and for the reviewers’ comments concerning our manuscript entitled “Network pharmacology and molecular docking reveal the effective substances andactive mechanisms of Dalbergia Odorifera in protecting against ischemic stroke” (Manuscript Nummber PONE-D-21-05118). The comments are valuable and have been very helpful for revising and improving our paper, as well as guiding the importance and significance of our research. We have studied the comments carefully and made corrections, and hope that it will be met with approval. Revised portion are marked in red in the manuscript.

**Replies to Reviewer 1**

**Q: The present paper will be certainly more exhaustive for the audience of readers of this magazine if the authors will confirm, in an adequate in vivo model of cerebral ischemia, the efficacy and the effective dosage of the extracted drugs from Dalbergia Odorifera.**

**A:** Thank you for your suggestions. MOL002974 (butein) isolated from Dalbergia odorifera is an important core bioactive component screened by network pharmacology and molecular docking. In order to evaluate the cerebral protection of butein, we replicated the middle cerebral artery occlusion (MCAO) model in rats to detect the effects of different doses of MOL002974 (butein) on neurological score, infarct volume and lactate dehydrogenase (LDH) level of MCAO rats. The results indicated that MOL002974 (butein) could significantly improve the neurological score of rats, decrease cerebral infarction volume, and inhibit the level of LDH in a dose-dependent manner. Details have been added to the manuscript.

**Replies to Reviewer 2**

**Q1:** **In ”methods” section, it is necessary to explain the** **rationale of dose choose of** **MOL002974, MOL002975, and MOL002914 providing information relating to preliminary data.**

**A:** Thanks for your constructive comments. In order to choose the appropriate dosage, CCK8 method was used to detect the effects of different concentrations of 3 core bioactive components (MOL002974, MOL002975 and MOL002914) on the survival rate of PC12 cells of oxygen glucose deprivation/reperfusion (OGD/R). The results have been submitted as supplementary material.

**Q2: The authors demonstrated that there was a significant increase of p-AKT expression, after treatment, for 24 hours, of PC12 cells incubated with MOL002974, MOL002975, and MOL002914. It would be interesting to show the effects of these components on cell survival.**

**A:** Thank you for your suggestions. The CCK8 method was used to detect the effects of 3 core bioactive components on cell viability. The cell viability results showed that MOL002974, MOL002975, and MOL002914 all improved the cell survival rate in a dose-dependent manner and alleviated the damage to PC12 cells in the OGD/R group, suggesting that the 3 core bioactive components could all promote cell survival. In addition, MOL002974 had the best effect on improving cell survival when the 3 core bioactive components are at the same concentration, suggesting that MOL002974 played a more important role in improving cell survival than the other 2 core bioactive components. Details have been added to the manuscript.

We would like to express our sincere thanks to the editors and reviewers for their constructive and positive comments. We are looking forward to hearing from you.

Thank you.

Best regards,

Miaomiao Xi
